# Study on dynamic characteristics of salinized silt under cyclic loading

**DOI:** 10.1371/journal.pone.0290648

**Published:** 2024-03-06

**Authors:** Biao Wu, Jing Yuan Kou, Ming Min Xuan, Yu Li, Xi Yong Xu, Wen Ni Yi

**Affiliations:** 1 China Airport Construction Group Corporation, Beijing, China; 2 School of Civil Engineering, Central South University, Changsha, Hunan, China; 3 Shandong Provincial Airport Management Group Corporation LTD, Jinan, Shandong, China; Shandong University of Technology, CHINA

## Abstract

Under the repeated action of aircraft taxiing load, the subgrade plastic deformation becomes the key factor affecting the service performance of the airfields when salinized silt is used to fill the subgrade. In this study, the dynamic triaxial tests were carried out on a region in the northern part of China to study the effects of different salt contents on the dynamic characteristics of silt under cyclic loading. A prediction model for the salinized silt dynamic strength with a plastic strain of 4% as the failure criterion for the subgrade was thus proposed. It is found that with the increase of dynamic stress amplitude, the salinized silt plastic deformation transforms gradually from plastic deformation to incremental failure. The salt contents significantly influence the plastic strain and critical dynamic stress of silt. The strength of the salinized silt specimen is related to the ion concentration in the soil pores and the arrangement pattern of soil particles, as indicated by the progressive strength increase of the salinized silt at the low salt content of 1% and a further gradual decrease at high salt content.

## Introduction

Silt is widely distributed in North and Northwest China, where freshwater resources are relatively scarce. Therefore, saline in inland lakes can be considered for filling the airfield subgrades, which inevitably causes silt salinization. However, silt is characterized by loose structure and easy liquefaction. Under the long-term action of aircraft loading, the service performance of salinized silt subgrade remains unclear [1].

In order to explore the dynamic characteristics of subgrade (roadbed) under long-term cyclic loading, many researchers have studied the critical dynamic stress characteristics and the development trend of plastic strain under dynamic action. Larew and Leonards [2] first proposed the critical dynamic stress to describe the dynamic characteristics of soil, and the maximum cycle ratio of its lossless structure was recognized by several researchers. Tang et al. [3] analyzed the pore pressure and axial plastic strain of roadbed soil and determined the critical dynamic stress according to the failure depth. This method could not directly and accurately obtain the critical dynamic stress. Wang et al. [4] studied the influence of plastic strain of coarse-grained soil filler on railway subgrade under different states and proposed a calculation model for critical dynamic stress applicable to railway subgrades. Nie et al. [5] studied the dynamic characteristics of coarse-grained soil under heavy-haul railway loads and proposed a method of representing the plastic deformation rate by the ratio of critical dynamic stress to the strain growth rate. Li et al. [6] studied the plastic deformation characteristics of coarse aggregates in highway subgrades to explore the influence of dynamic stress amplitude and cyclic vibration frequency on plastic deformation. However, due to the periodicity and protracted nature of traffic load and the complexity of the basic physical properties and mechanical conditions of the soil, the findings on coarse-grained soils cannot be applied to silt roadbeds. Some researchers also studied the dynamic characteristics of saturated silt. Hussain et al. [7] studied the saturated silt dynamic characteristics in seismically active areas of Gujarat, India, and demonstrated that the cyclic strength rapidly decreased with the decrease of plasticity index and the fine particle content. Tong et al. [8] studied the dynamic characteristics of undisturbed and remolded marine silt through cyclic dynamic triaxial tests and concluded that the difference between undisturbed and remolded marine silt in dynamic strength decreased with the increase of consolidation ratio under the same equivalent cyclic vibration frequency.

Until now, few studies have been done on the dynamic characteristics of unsaturated silt under cyclic traffic loading. Zhang et al. [9] analyzed the strain growth rate of frozen silt in the subgrade and proposed critical dynamic stress models under different states. Nguyen et al. [10] investigated the influence of the high cyclic stress ratio on the dynamic parameters of kaolin and showed that the kaolin content significantly affected the soil dynamic characteristics. Wang et al. [11] studied the plastic strain of unsaturated silt and proposed a plastic strain model considering multiple factors. However, the effects of critical dynamic stress and salt content in silt were not studied in this study. Therefore, the dynamic characteristics of unsaturated silt under cyclic loading need further investigation. There have been few studies reported on critical dynamic stress and silt salinization.

Silt salinization results in a change in soil behavior and properties. Many researchers have studied the effect of salt content on soil properties. Ridley et al. [12] found that the optimum moisture content (OMC) of soil gradually decreased with the increase of salt content by adding saline water. Through consolidation tests, Yin et al. [13] confirmed that salt in pore solution increased the initial and final creep deformation of soil. Zhang et al. [14] and Xu et al. [15] conducted shear tests and triaxial compression tests on loess soils prepared with different salt contents, respectively, and found that with the increase of chloride salt content, the shear strength parameter of soil decreased first and then increased. Han et al. [16] conducted freeze-thaw cycle tests and unconsolidated undrained triaxial compression tests on remolded saline soil and revealed that the peak values for cohesion and undrained shear strength significantly decreased with the number of freeze-thaw cycles. However, the aforementioned studies were based on conventional static load. At present, a few studies have been conducted to explore the relationship between salt content and dynamic strength based on dynamic triaxial tests. Through the dynamic triaxial tests, Li et al. [17] found that when the vibration frequency reached 100, the higher the soluble salt content, the lower the saline soil dynamic strength. Through the dynamic triaxial and freeze-thaw tests, Zhang et al. [18] found that the initial dynamic modulus damage of silt increased linearly with the increase of salt content. However, the above studies did not logically relate the influence of salt content on the soil to the critical dynamic stress and plastic strain or express it through formulas.

Therefore, in order to explore the influence of salt content on the silt dynamic characteristics in the subgrade, dynamic triaxial tests were carried out in this study on silt under different dynamic stress amplitudes and salt contents, revealing the influence trend of salt content on the dynamic characteristics of silt and proposing the plastic strain model for salinized silt and the prediction model of critical dynamic stress for salinized silt under the stable plastic limit state. The critical dynamic stress of the salinized silt was also analyzed, and the internal cause of dynamic response evolution was explored, providing a basis for designing railway and airfield subgrades in regions with salinized silt.

## Soil properties and test scheme

### Test instruments

**Fig 1:**
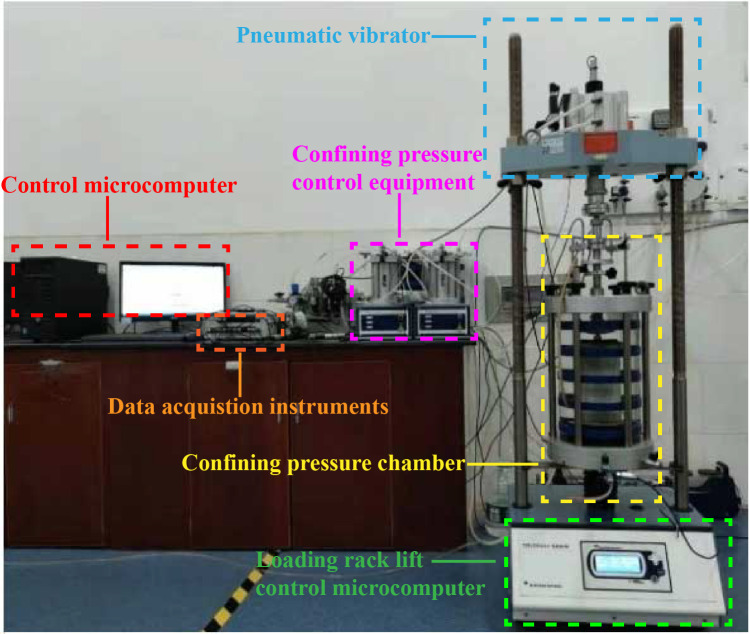
Dynamic triaxial test system.

The test instrument in this study was Controls/WF static and dynamic triaxial test system manufactured by Britain (Fig 1), with the type Dynatriax100/14, which has both the test system and data acquisition system. The specimen was formed by a self-made compaction sampler (the hammer weight was 2.5 kg, and the drop height of the hammer head was 45 cm). The count of hammer blows of each layer was kept constant during specimen preparation. An automatic demolding instrument was used to reduce the disturbance caused to the specimen during demolding.

### Test soil properties

**Fig 2:**
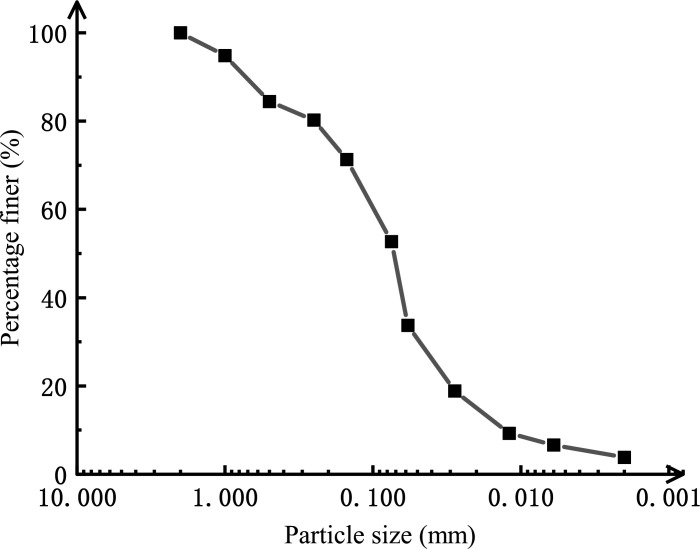
Particle size distribution of silt in the study area.

In this study, the silt specimens were taken from a 5 m depth of the construction area for the subway of an airfield pavement in northern China. From the underground water level survey, the silt within 5 m below the surface of the construction area is unsaturated all year round. According to the test based on the code [19] (GB/T 50123-2019), the test silt is sandy silt, with a plastic limit of 15.4%, liquid limit of 23.0%, plastic limit index of 7.6, a specific gravity of particles of 2.67, the optimum moisture content (OMC) of 13.1%, and maximum dry density of 1.87 g/cm^3^. The grading curves for soil are shown in Fig 2, and the basic soil properties of the silt are shown in Table 1.

**Table 1:** Physical properties of silt.

Properties	Optimum moisture content (%)	Specific gravity of soil particle	Plastic limit (%)	Liquid limit (%)	Plasticity index	Internal friction angle(°)	Cohesion(kPa)	Void ratio	Maximum dry density(g/cm^3^)
**Values**	13.1	2.67	15.4	23.0	7.6	28.8	50.5	0.52	1.87

**Fig 3:**
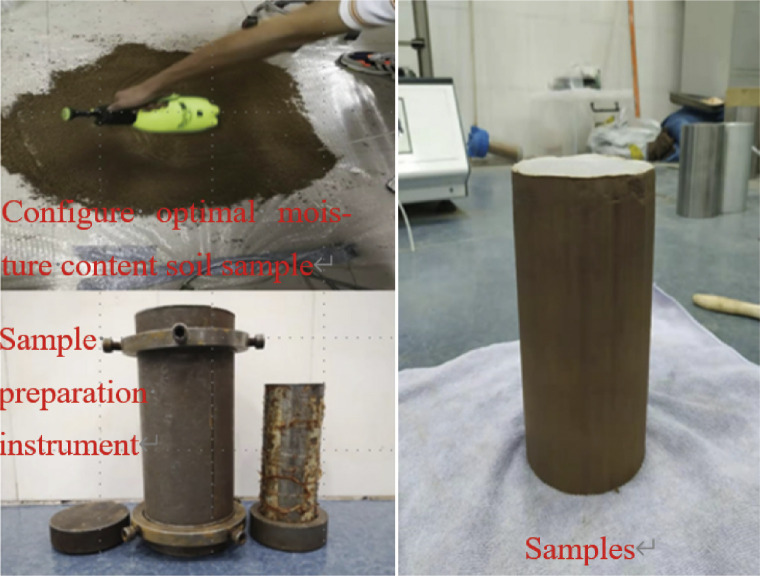
Schematic of soil sample preparation.

The strength characteristics of undisturbed and remolded soils have been studied by some researchers [20], and it has been found that the strength of undisturbed soil was slightly higher than that of remolded soil. Similarly, the strength of undisturbed soil was slightly higher than that of remolded soil under dynamic strength. Therefore, the soil used in this study is the remolded soil, arranged according to the properties of undisturbed soil and the actual engineering scenario. After site sampling, the soil was dried, crushed, and sifted. According to the OMC of the soil, deionized water was used to prepare 1%, 3%, and 5% sodium chloride solutions. The solution was sprayed evenly into the dried soil and left there for 48 hours after being stirred evenly. A 100 mm diameter and 200 mm high mold was used for preparing compacted specimens (Fig 3). The specimens were molded into five layers for compaction, and the surface of each layer was scraped after compaction. After the specimens were molded, they were wrapped in plastic and left at room temperature for 24 h. Silt with OMC is usually used to fill the field to ensure the compaction effect. A large subgrade area was hardened and covered, and the moisture content did not change significantly within the soil depth range in response to the aircraft load. Therefore, in this work, unsaturated soil with a moisture content of 13.1% (a saturation level of 67.4%) was selected for related tests.

### Design of test scheme

To the analysis of the confining pressure of silt at the field subgrade depth of 1- 5 m, the confining pressure of soil is approximately in the range of 30–90 kPa according to the code [21] (MH/T 5010-2017), which considering the thickness of the pavement structure and the bulk density of the silt in the subgrade. The confining pressure of 60 kPa was used to investigate the plastic strain development law of the silt in the literature [22]. In this paper, the same confining pressure of 60 kPa was selected to qualitatively study the dynamic characteristics of the salinized silt in the subgrade. According to the code specifications [23] (MH/T 5027-2013), the relative compaction degree (RC) within 0.8 m below the pavement in the filling section shall not be less than 96%, that within 0.8 m below the excavation section shall not be less than 94%, and that within below the filling section shall not be less than 92%. Considering the likelihood of uneven compaction in practical engineering works, the specimen compaction degree was uniformly taken as 94% (dry density of 1.7578 g/cm^3^) to conduct consolidated undrained tests.

During taking off and taxiing, the vibration frequency of the aircraft is related to its taxiing weight and traveling speed [24]. The regional aircraft vibration frequency is about 1 Hz, and the aircraft vibration frequency during taxiing is about 0.5-5 Hz. As the taxiing speed increases, the aircraft lift force increases, and the impact of aircraft dynamic load on the subway decreases [25]. The lower the frequency of the dynamic triaxial instrument, the more accurate the measurement data. Therefore, considering the aircraft vibration frequency and instrument accuracy comprehensively, the load frequency was taken as 1 Hz in this study, and half-sine wave mode was used to load the dynamic stress amplitude [26]. The maximum dynamic stress amplitude of the top surface of the subgrade is about 100 kPa during aircraft taxiing. To investigate the soil deformation characteristics under different dynamic stress amplitudes, the dynamic stress amplitudes are taken as *σ*_*d*_ =60, 80, 100, 120, 140, 180, 220, 240, and260 kPa, respectively. In the test, plastic strain reaching 10% or cyclic vibration frequency reaching 10,000 times were taken as the conditions for stopping the test [25,26].

**Fig 4:**
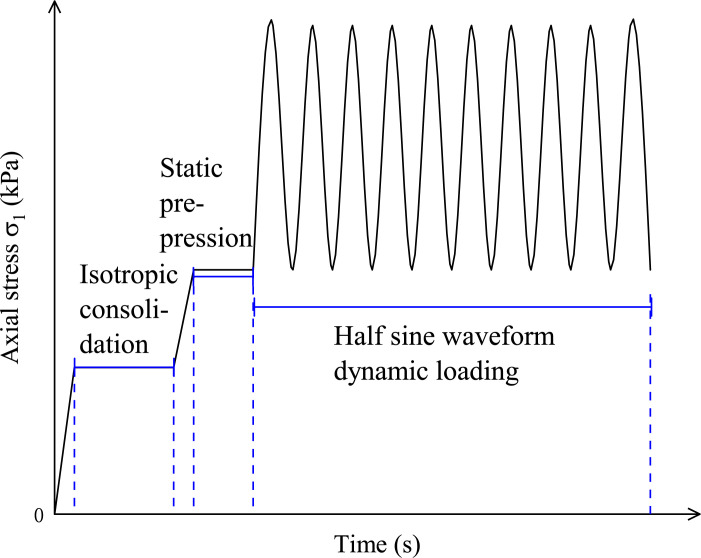
Schematic diagram of axial loading.

The test was divided into three loading stages, i.e., isobaric consolidation, static preloading, and dynamic stress application (i.e., half-sine wave loading) stages. Before dynamic loading, the static preloading was carried out for 100s with the compression of 15 kPa [20] (with the diagram of axial loading shown in Fig 4) to eliminate the irregularity in the upper and lower parts of the specimens and prevent the specimens undergoing excessive initial deformation of the specimens under sudden external force.

Since the salt content in undisturbed silt was lower than the limited salt content of weakly saline soil (0.3%), it can be determined that the silt specimens do not belong to saline soil, and the salt content in silt specimens is considered zero. The salt in the northwest area with salinized silt is dominated by chlorine salt, and the salt content in the shallow area of the airfield varies in the range of 0 and 5% [27]. Thus, the specimens with salt contents of 1%, 3%, and 5% (ratio of the mass of added sodium chloride to that of dried soil) were prepared to study the chloride influence on the dynamic characteristics of the subgrade with undisturbed silt. During consolidation, the specimen displacement was set to zero, and the salt content in the specimens before and after consolidation remained constant. A total of four groups of specimens (30 specimens) are designed to explore the dynamic characteristics of salinized silt under different salt contents and dynamic stress amplitudes, with the test scheme shown in Table 2.

**Table 2:** Test conditions of salinized silt.

The salt content *ω* (%)	The dynamic stress amplitude σ _d_ (kPa)
0	60, 80,100, 120, 140, 180, 220, 240
1	60, 80, 100, 120, 140, 180, 220, 240, 260
3	60, 80, 100, 120, 140, 160, 180
5	60, 80, 100, 120, 140

## Analysis of test results

Plastic strain is the intuitive reflection of subgrade settlement caused by the dynamic effect of salinized silt. The plastic strain and the critical dynamic stress represent the capacity of the subgrade to resist deformation and bear the dynamic load, respectively. In order to explore the internal factors for the dynamic response of salinized silt, the relationship between and critical dynamic stress and salt content in salinized silty soil was studied.

### Plastic strain analysis of salinized silt

**Fig 5:**
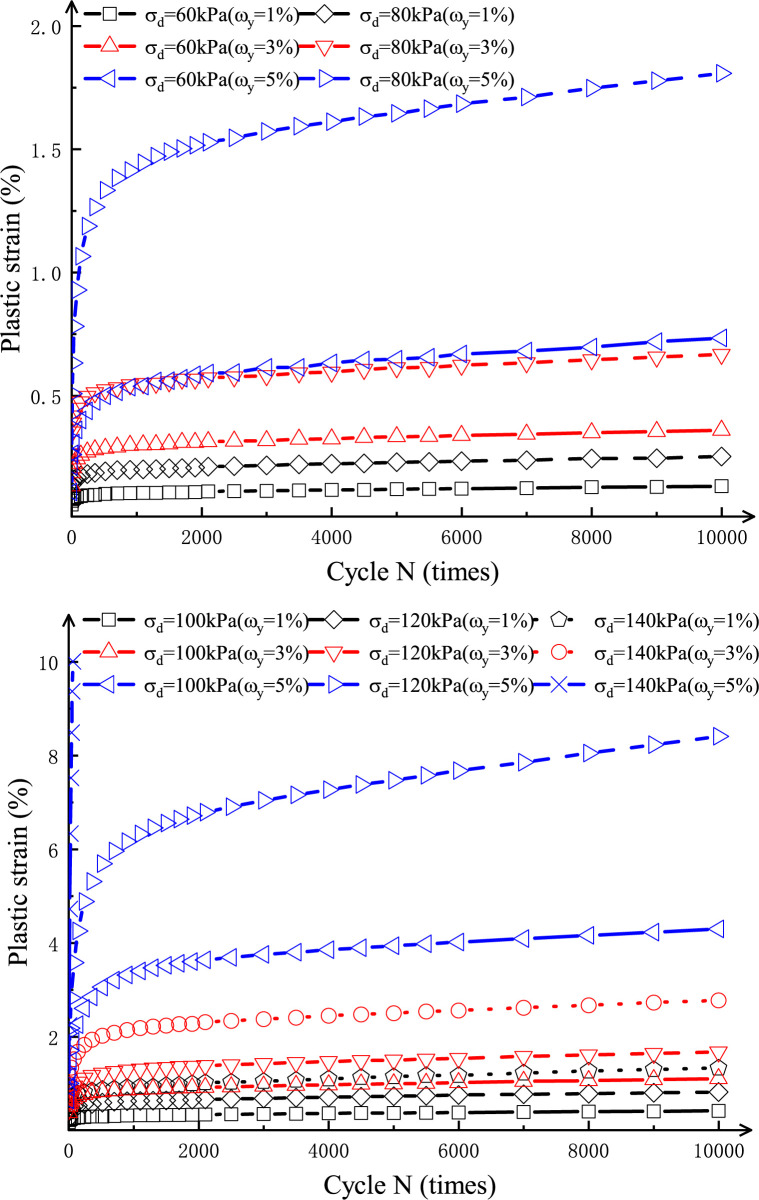
Relation curve between plastic strain and cyclic vibration times of salinized silt sample(RC=94%, σ_3_=60 kPa).

Fig 5 shows the variation of plastic strain ε_p_ with cyclic vibration frequency *N* of salinized silt specimens with salt content **ω**_y_ of 1%, 3%, and 5% under the dynamic stress amplitude *σ*_*d*_ of 60 kPa and 80 kPa at the relative compaction degree (RC) and confining pressure (*σ*_*3*_) of 94% and 60 kPa, respectively. According to Fig 5, the plastic strain increases with the increase in salt content and dynamic stress amplitude, and the salt content **ω**_*y*_ and dynamic stress amplitude *σ*_*d*_ significantly influence the development of plastic strain *ε*_*p*_. Under the same dynamic stress amplitude, the higher the salt content, the larger the plastic strain. The plastic strain increases rapidly when the dynamic stress amplitude reaches 140 kPa, with no stable state. Through analysis, the reasons are as follows: with the increase of salt content, the ion concentration in the pore solution increases, thus reducing the thickness of the double electric layer between soil particles and leading to the decreased repulsion force and strengthened bonding between particles. When the salt content is 0-1%, the stress between particles is larger, and the mobile range of particles is decreased, thus leading to the decreased plastic strain of the soil specimens. When the salt content is 1-5%, the layout of soil particles transforms from a directional dispersion structure to a directionless flocculation structure with the further increase in ion concentration of the salt solution. This phenomenon is due to the limit of the thickness of the double electric layer between soil particles, resulting in a loose soil structure. This is manifested by the increased plastic strain of soil under the same dynamic stress amplitude [28]. When *σ*_*d*_ is small, the plastic strain increases with the increase of the cyclic vibration frequency *N* and eventually tends to be stable. With the increase in *σ*_*d*_, the specimen plastic strain at the early loading stage develops rapidly; with the increase of *N*, the growth rate of plastic strain decreases to some extent but still presents an increasing trend. Therefore, the growth trend on soil plastic strain under cyclic loading is significantly affected by the dynamic stress amplitude, primarily due to cyclic dynamic load and critical dynamic stress, i.e., the maximum cyclic stress of soil without structural failure, existing in the structural deformation of soil. It can be used to describe the permanent deformation characteristics of granular materials such as subgrade filler under cyclic loadings [27].

**Fig 6:**
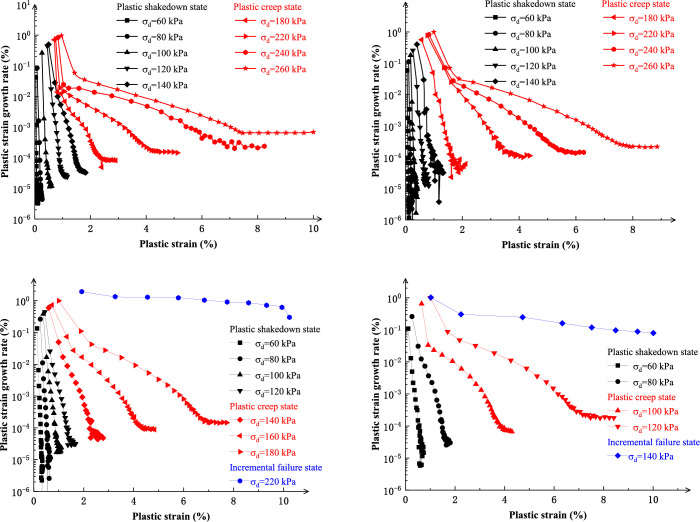
Relationship curve between plastic strain development rate and plastic strain under different salt content.

Fig 6 shows the relation curves between the plastic strain development rate and the plastic strain in the test process of salinized silt specimens. The curves are divided into different sections [5]. Werkmeister et al. [29] divided the plastic deformation behavior of granular material under cyclic loading into three types, i.e., plastic deformation behavior, plastic creep behavior, and incremental failure behavior. Compared with Fig 5, it can be seen that when *σ*_d_ is smaller, the plastic strain generated by the specimens in the whole cyclic loading process is smaller, and the strain is less than 2%. At the early loading stage, the specimen plastic deformation develops faster, and with increased cycles, the strain growth rate decreases gradually. When the loading reaches a certain number of cycles, the specimens enter the stable loading stage, and the plastic strain is in a relatively stable or slightly growing state. In this case, specimen plastic strain is manifested as a plastic deformation. With the increase of *σ*_*d*_, specimen plastic strain develops rapidly in the early loading stage, and the dynamic stability of the specimen is broken. In the late stage of cyclic loading, the plastic strain increases continuously at a certain growth rate. Although the specimen structure is in a relatively safe state, it is still likely that with the further increase of the cyclic vibration frequency, the plastic strain rate changes abruptly, and then rapid structural failure occurs. In this case, plastic deformation is manifested by plastic creep. When *σ*_*d*_ exceeds a certain limit, larger deformation begins in the test at the beginning of the cycle, and the growth rate of plastic deformation is larger. After limited cyclic vibration frequency, the specimens undergo structural failure due to excessive plastic deformation. In this case, the specimens’ failure mode is generally manifested as compression expansion and shear failure, and the specimen strain behavior is manifested as an incremental plastic failure.

When the salt content is 3% and 5%, the failure modes of specimens vary greatly under different dynamic stress amplitudes, and structural failure occurs rapidly when the dynamic stress amplitudes are 220 kPa and 140 kPa, respectively. For the specimens with salt contents of 0% and 1%, when the dynamic stress amplitude is 260 kPa, their strain rates all increase, whereas these are still in the plastic creep stage. The strain rate of specimens with a salt content of 0% grows faster, reaching 10% when the test stops. If the critical state between plastic creep and incremental failure is taken as the criterion for structural failure of soil when the dynamic stress amplitude is 260 kPa, the specimens with a salt content of 0% are close to structural failure, while the strain of the specimens with the salt content of 1% grows in a relatively steady way.

(a)Salt content **ω**_y_=0% (b)Salt content **ω**_y_=1%
(c)Salt content **ω**_y_=3% (d)Salt content **ω**_y_=5%

**Fig 7:**
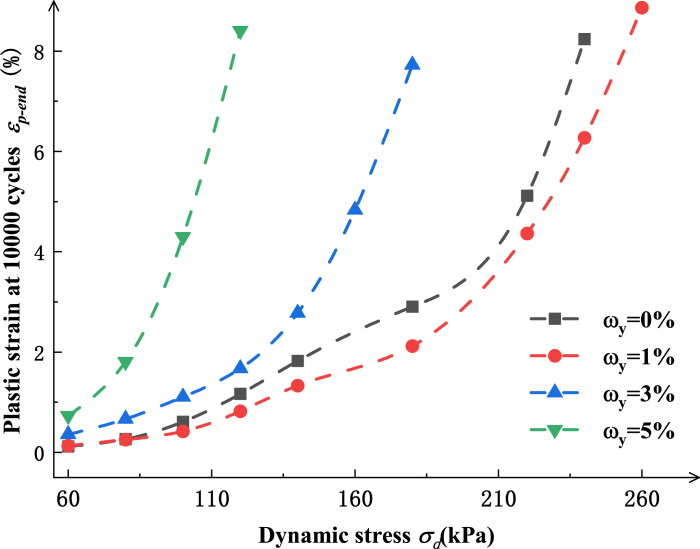
The plastic strain *ε*_p-end_ -*σ*_d_ curve at different salt contents when the test stops (*N*=10000, RC= 94%, σ_3_=60 kPa).

Fig 7 shows the variation curve for the plastic strain with the dynamic stress amplitude of specimens with different salt contents when the test stops (*N*=10000 times) under confining pressure *σ*_*3*_=60 kPa. As shown in the Fig 7, the influence of salt content on specimen plastic strain goes through two stages. The first stage is the transition from undisturbed silt (with a salt content of 0%) to saline silt (with a salt content of 1%). In this stage, the increase of salt content contributes to the increase in the specimen “hardness”. Under the same dynamic stress amplitude, the plastic strain of saline silt is smaller than that of undisturbed silt. The second stage is the development stage from a low salt content to a high salt content. In this stage, the “hardness” of the specimens decreases rapidly with the increase of salt content. Under the same dynamic stress amplitude, the higher the salt content, the larger the plastic strain of the specimen.

In practical engineering applications, the roadbeds usually do not suffer sudden failure under a limited traffic dynamic load. However, the traffic loads produce a larger settlement in long-term service, leading to pavement structure failure and making the runway unserviceable. This results in the case that the runway cannot continue to be in service. Therefore, determining the critical dynamic stress level between plastic deformation and plastic creep states of subgrade silt is a key factor in judging whether subgrade silt can achieve the dynamic stability state under traffic load.

### Plastic strain model of silt considering salinization

According to the exponential model proposed by Monismith et al. [30], the *ε*_*p-end*_*-σ*_*d*_ relation for salinized silt with different salt contents under the same confining pressure is shown in Eq (1), where *A*_1_ and *B*_1_ are parameters related to soil properties:

$$\varepsilon_{p‐\mathrm{end}}=A_{1}(e^{B_{1}*\sigma_{d}}-1)$$ (1)

**Fig 8:**
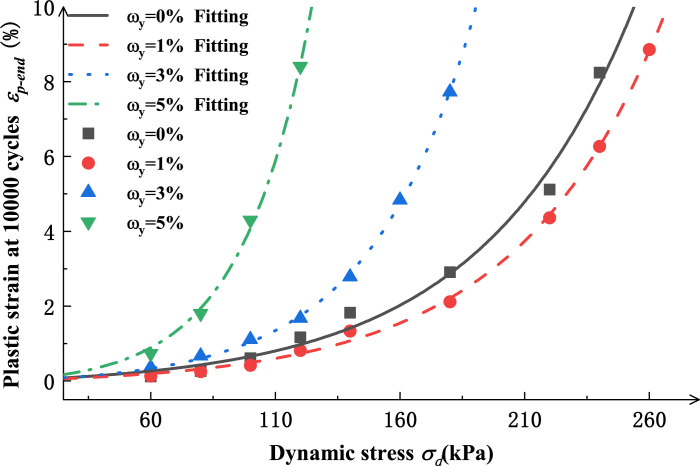
Fitting curves of *ε*_p-end_ -*σ*_d_ relation of salinized silt under different confining pressures (RC= 94%, *σ*_*3*_=60 kPa).

The model in Eq (1) is used to calculate the *ε*_*p-end*_*-σ*_*d*_ relation curves for salinized silt under different salt contents in Fig 7, as shown in Fig 8. Table 3 shows the parameter results.

**Table 3:** Parameter values of ε_p-end_-N relation formula of salinized silty soil with different salt contents.

The salt content ω (%)	The confining pressure σ_3_ (kPa)	*A*_1_	*B*_1_	*R* ^2^
0	60	0.1582	0.0164	0.98
1	60	0.1120	0.0169	0.99
3	60	0.1022	0.0241	0.99
5	60	0.1177	0.0358	0.99

It can be seen that when the salt content increases from 0 to 5%, *A*_1_ first decreases and then increases with the increase of salt content *ω*_*y*_, with the fitting relation given in Eq (2); parameter *B*_1_ rapidly increases with the increase of salt content *ω*_*y*_, with the fitting formula shown in Eq (3).


$$A_{1}=\frac{0.0426}{{\omega_{y}}^{0.264}}\;R^{2}=0.90$$ (2)

$$B_{1}=0.0187-0.318\omega_{y}+8.51{\omega_{y}}^{2}\;R^{2}=0.99$$ (3)

By substituting Eqs (2) and (3) into Eq (1) and sorting out the result, Eq (4) for the variation of plastic strain *ε*_p-end_ with dynamic stress amplitude *σ*_*d*_ and salt content *ω*_*y*_ in salinized silt with different salt contents when the cyclic vibration frequency *N*=10000 times can be obtained.


$$\varepsilon_{p‐\mathrm{end}}=A_{1}(e^{B_{1}*\sigma_{d}}-1)=\frac{0.0426}{{\omega_{y}}^{0.264}}\times (e^{(0.0187-0.318\omega_{y}+8.51{\omega_{y}}^{2})*\sigma_{d}}-1)$$ (4)

### Failure criteria for critical dynamic stress of salinized silt

It has been suggested that the abrupt point of deformation curvature under dynamic load should be taken as the criterion for identifying soil failure strain [31]. Through laboratory dynamic triaxial test, Lee et al. [32] found that when the strain of clay with high sensitivity reaches 4%-6% and that of clay with low sensitivity reaches 2%-3%, the failure of the shear plane will occur. In this case, if the cyclic vibration frequency continues to increase, the strain will rise rapidly, thus leading to the instability of specimen structures. In order to make the unified determination of soil structure failure, the single strain of 3% was proposed as the standard for judging soil failure strain. Seed et al. [31] took the specimen plastic strain reaching a certain value (such as 5% or 10%) as the failure strain standard. In addition, according to the engineering background of soil specimens, many researchers took the plastic strain of 1%, 5%, 10%, or 15% as the standard for failure strain [30]. The soil failure criteria can be divided into two types – one is the failure of soil structure in the test process as the failure strain standard, and the other is to calculate the maximum settlement that soil can bear according to the engineering design conditions and take this conversion as the failure strain standard. From the engineering perspective, the roadbed “failure” is not the structural failure of roadbed soil in the real sense but whether the roadbed can still meet the normal traffic operation conditions. Therefore, comprehensively considering the actual engineering situation of runway subgrade and the development characteristics of specimen strain in the tests, according to the specification of foundation settlement index in the code (MH/T 5027-2013), the allowable settlement deformation of subgrade soil in the runway under moving load can be calculated as 4%-6%.

In addition, by analyzing the *ε*_*p-end*_*-σ*_*d*_ curves for specimens with different salt contents (in Fig 7), it is found that the growth rate of plastic strain accelerates when the specimen plastic strain *ε*_p-end_ reaches 2%-4%. It tends to be stable afterward, presenting a linear growth trend. In combination with the analysis of soil failure criteria, the plastic strain *ε*_*p-end*_ reaching 4% can be regarded as the separation point for *ε*_p-end_*-σ*_*d*_ variation curves under different test conditions. In this paper, the plastic strain reaching 4% has been taken as the failure strain standard, and the cyclic vibration frequency corresponding to the specimen strain reaching 4% during the plastic strain development for different specimens is taken as the cyclic vibration frequency *N*_*tp*_ for specimen failure.

### Prediction model for critical dynamic stress of salinized silt

Eq (4) is used to calculate the dynamic stress amplitude required when the plastic strain of salinized silt specimens with different salt contents reaches 4% when the cyclic vibration frequency *N*=10000 times, as given in Table 4.

**Table 4:** Dynamic stress amplitude required for the cumulative axial strain to reach 4% at N=10000 times for the sample with different salt content.

The salt content*ω* (%)	The dynamic stress amplitude σ_d_ (kPa)
1	213
3	153
5	97

**Fig 9:**
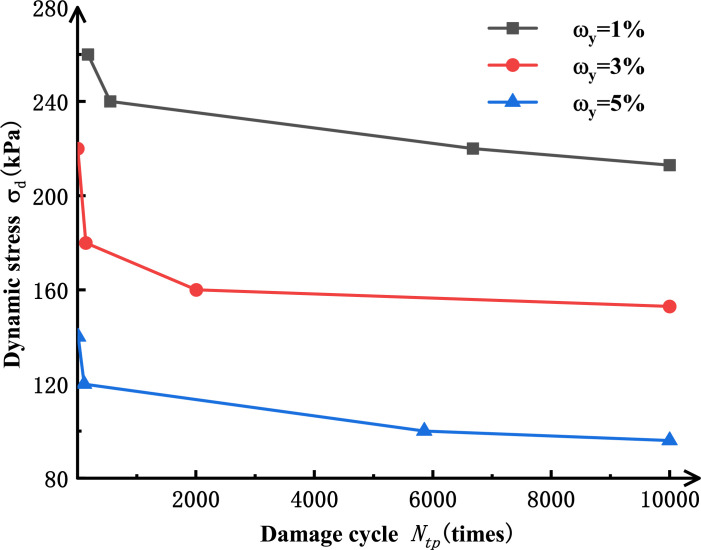
The dynamic stress amplitude-cyclic vibration time curve of silt with different salinity reaches failure strain (σ_*3*_=60 kPa).

Fig 9 shows the relationship between dynamic stress amplitude and cyclic vibration frequency when salinized silt specimens with different salt contents reach failure strain. As shown in Fig 9, when the standard for soil failure strain is reached, the larger the salt content, the smaller the required dynamic stress amplitude.

The relationship between dynamic stress *σ*_d, tp_ and the cyclic vibration frequency *N*_tp_ of corresponding failure when silt reaches failure strain under different salt contents can be described by Eq (5) [33].


$$\sigma_{d.tp}=\alpha_{0}+\beta_{0}*{\gamma_{0}}^{N_{\mathrm{tp}}}$$ (5)

Where *α*_0_, *β*_0,_ and *γ*_0_ all denote fitting parameters, with the fitting values presented in Table 5.

**Table 5:** Fitting parameter values.

The salt content *ω* (%)	*α*_0_	*β*_0_	*γ*_0_	*R* ^2^
1	213.0967	60.4267	0.9985	0.95
3	144.3426	59.2280	0.9994	0.97
5	95.0004	48.1896	0.9943	0.78

**Fig 10:**
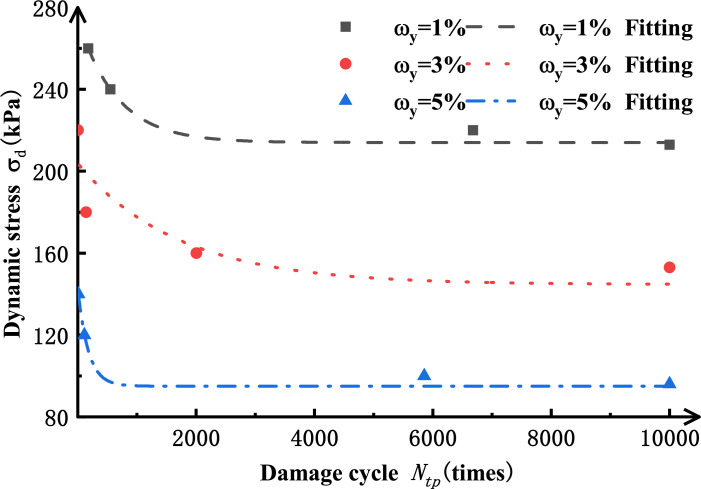
The dynamic stress amplitude-cyclic vibration fitting curve of remolded siltunder different salt content reached the failure strain(σ_3_=60 kPa).

**Fig 11:**
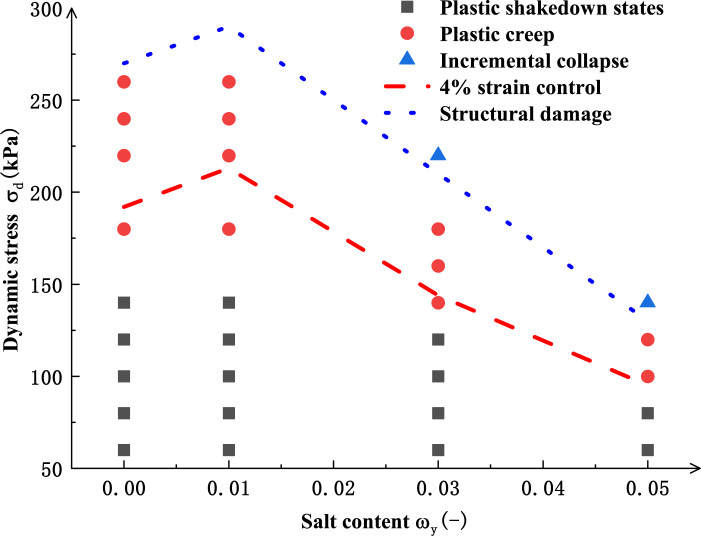
Critical dynamic stress curves of samples with different salt content.

The fitting results are shown in Fig 10. As shown in Fig 10, Eq (5) has a better fitting effect on the dynamic stress-cyclic vibration frequency curve as the salinized silt specimens reach failure strain when *σ*_3_=60 kPa under different salt contents. As seen from the values for fitting parameters in Table 4, in Eq (5), *σ*_*d*, *tp*_ gradually decreases with the increased failure vibration frequency *N*_tp_. When the failure cyclic vibration frequency *N*_tp_ approaches infinity, *σ*_d, tp-min_=*α*_0_. Similarly, for salinized specimens under different conditions, when the loaded dynamic stress amplitude is smaller than *α*_0_, with the increase of cyclic vibration frequency, the specimen plastic strain will tend to be a stable value, less than 4%. Therefore, it can be considered that the critical dynamic stress of plastic deformation should be the minimum value for dynamic stress *σ*_d, tp-min_ when the specimens reach failure strain. Therefore, Table 6 shows the values for critical dynamic stress of plastic deformation of silt specimens with different salt contents. Fig 11 shows the critical dynamic stress curves for the specimens with different salt contents.

**Table 6:** Critical dynamic stress amplitude of plastic stability of salinized silt samples with different salt contents.

*ω*/%	σ_3_/kPa	σ _d,tp_ /kPa
0	60	192
1	60	213
3	60	144
5	60	95

The relationship between salt content and dynamic stress amplitude is divided according to the classification standard of specimen deformation behavior, with dotted lines used to distinguish different deformation behaviors. Fig 11 shows that critical dynamic stress presents similar development trends under different failure criteria. Also, the salt content significantly influences the critical value of the plastic deformation state of salinized silt. The higher the salt content, the smaller the critical value for plastic deformation. In practical engineering, subgrade failure refers to whether the subgrade can meet the needs of normal operation rather than structural failure in a real sense. Therefore, the critical dynamic stress value is smaller when the strain of 4% generated by the specimens is taken as the failure standard in this paper, which is more in line with engineering practice. The critical value for plastic deformation of soil with a salt content of 1% is about 2.2 times that of soil with a salt content of 5%. When the salt content is 0%-1%, due to the lower ion concentration in the pore solution, the winged colloid is formed on the soil particle surface, and the bonding between particles is strengthened [34], resulting in the enhanced critical dynamic stress of soil. When the salt content is 1%-5%, the winged colloid gradually disappears, the distribution range of pores inside the soil becomes larger, and a loose flocculating structure is formed inside the soil [28], resulting in the loose soil structure and leading to a sharp reduction in the critical dynamic stress. When the salt content is 5%, the critical dynamic stress decreases by about 60%.

From the variation trend of the plastic strain and critical dynamic stress of salinized silt, it is observed that with the increase of salt content, the specimen plastic strain increases significantly compared with the undisturbed silt specimens. However, the corresponding critical dynamic stress decreases considerably due to the bonding of soil particles involving contact connection, adsorption water film connection, and cementation connection between soil particles [28]. With the increase of salt content, the ion concentration in pore water increases, resulting in the change of internal structure in the soil, resulting in the significant degradation of soil stiffness and strength.

## Conclusions

In this paper, large-scale dynamic triaxial tests were carried out on remolded silt with different salt contents to analyze the influence of salt content on the subgrade silt characteristics, such as the plastic strain development, the critical dynamic stress, etc. And in combination with the dynamic triaxial tests, the prediction model of plastic strain and critical dynamic stress for saline silt considering salt contents are obtained. The main conclusions are as follows:

1. Salt content significantly affects the silt dynamic characteristics under dynamic load. The plastic strain and dynamic stress of the salinized silt trend to decrease and then increase at the limit of 1% salt content as the salt content increased from 0 to 5% for the same dynamic stress amplitude. The rate of increase in plastic strain increases with increasing salt content. A high salt content leads to a low dynamic stress amplitude for the plastic creep state and incremental failure state. A model for predicting the plastic strain of salinized silt considering the salt content is also proposed based on the test data, and the model can well predict the relationship between salt content and plastic strain.
2. Based on the plastic strain reaching 4% as the failure strain standard for the silt subgrade, a model for predicting the critical dynamic stress of salinized silt considering different salt content is proposed, and the salt content has a significant effect on the critical value for plastic deformation of salinized silt. A large the salt content results in a low the critical value for plastic deformation. The critical value for plastic deformation of specimens with a salt content of 1% is about 2.2 times that of salinized specimens with a salt content of 5%. The research in this paper can be used as reference for calculating load bearing capacity of the silt subgrade of the airfield with different salt contents.

## Supporting information

S1 Data (ZIP)
