# Correction: Study on dynamic characteristics of salinized silt under cyclic loading

**DOI:** 10.1371/journal.pone.0316844

**Published:** 2024-12-30

**Authors:** Biao Wu, Jing Yuan Kou, Ming Min Xuan, Yu Li, Xi Yong Xu, Wen Ni Yi

There is an error in affiliation 1 for authors Biao Wu and Jing Yuan Kou. The correct affiliation 1 is: China Airport Planning and Design Institute (Group) Co., Ltd., Beijing, China.
